# Radiolabelled monoclonal antibodies in the treatment of metastatic cancer

**DOI:** 10.3747/co.2007.112

**Published:** 2007-02

**Authors:** D.M. Goldenberg

**Keywords:** Antibodies, bi-specific antibodies, cancer, pre-targeting, radioimmunotherapy, radionuclides

## INTRODUCTION

Most cancer patients die from advanced, metastatic disease. Because surgery and external irradiation are the best prospects for treating localized disease (although both have also been applied to limited treatment of metastatic disease), systemic chemotherapy—and more recently, immunotherapy—are the mainstays of treating cancer that has become disseminated. Systemic chemotherapy is accompanied by dose-limiting toxicities to various organ systems, particularly the rapid proliferating cells of the oral and intestinal mucosa, bone marrow, and hair follicles, and agents that target antibodies are usually less potent, cause infusion or flu-like reactions, and are most effective in combination with cytotoxic drugs 1,2.

Another class of agents that combines the foregoing properties has emerged in the form of radiolabelled antibodies, which harness the ability of antibodies against cancer antigens to localize to cancer cells, and the cytotoxicity of the radiation carried by the antibodies. In 1978, I named this method, then in its infancy, “radioimmunotherapy” 3. The last quarter-century has witnessed development in all aspects of this therapy, such as choice of antibody and antibody form, selection of therapeutic radionuclide, conjugation chemistry, and tumour target and clinical setting. These aspects have been reviewed elsewhere 4–7, and so they will not be the principal focus of this article. Instead, I will summarize the status, including problems and prospects, for the application of radioimmunotherapy in the treatment of meta-static cancer.

## RADIOIMMUNOTHERAPY AND METASTATIC CANCER

The experience with radioimmunotherapy has been different for hematopoietic disease and for solid cancers, with non-Hodgkin lymphoma (nhl) therapy being the use for which the first radioimmunoconjugate products gained regulatory approvals and started into clinical use 8–11. The two approved products both consist of murine antibodies against CD20, with added naked antibody protein—either murine or chimeric (human/mouse) monoclonal antibodies (mAbs). At doses delivered to tumour of less than 20 Gy (usually around 10 Gy), therapeutic efficacy is superior to that of naked mAb counterparts 12,13 and even comparable to the effects of complicated regimens of cytotoxic drugs or to the use of naked mAbs in combination with chemotherapy, as reported for nhl patients with advanced disease 8–11. Those findings support the view that radioimmunotherapy can be effective if used in the proper setting and if sufficient radiation is targeted.

In the case of the less radiosensitive solid tumours, the best results have been seen in the settings of minimal disease or of compartmental, or regional, application so as to optimize the dose of radiation delivered. Many antibodies bearing a variety of radionuclides are being tested clinically against diverse cancers (Table i), and some have in fact shown encouraging targeting and some evidence of tumour response. However, only in the setting of minimal disease, such as an adjuvant setting, or with the use of an indirect method of radioimmunotherapy called “pre-targeting” has evidence of clinical efficacy been reported.

The adjuvant case involves the use of ^131^I-labelled, humanized, anti-carcinoembryonic antigen (cea) mAb after salvage resection of colorectal metastases to the liver, where a doubling of median survival to 68 months was reported during long-term follow-up 14. In the pre-targeting mode, the tumour is first targeted with a bi-specific antibody. One arm of the antibody binds to the tumour antigen (for example, cea); the second arm targets a peptide that is given in a second step to deliver the radioactivity to the tumour through binding to a tumour-localized antibody 7,15–17. In a study of patients with cea-expressing medullary thyroid carcinoma, improved survival was found in the patients receiving the ^131^I–peptide after pre-targeting when those patients had a high tumour-marker (calcitonin) doubling time; patients not given radioimmunotherapy or lacking the increased calcitonin doubling time showed significantly poorer outcome 18. Still other methods of pre-targeting, not involving bi-specific antibodies, have also shown evidence of therapeutic efficacy when given as compartmental therapy in brain cancers 19.

Finally, even directly radiolabelled antibodies have shown therapeutic promise in preclinical and clinical studies involving regional or compartmental administration, such as in brain cancers 20 and also in peritoneal spread of ovarian and colorectal cancers 21–23. However, as discussed elsewhere 24, a pivotal, randomized trial of adjuvant intraperitoneal radioimmunotherapy in ovarian cancer with a radio-labelled murine anti-Muc-1 mAb failed to show any efficacy advantage 25.

## CONCLUSION

The variety of studies and approaches are, in my view, encouraging, because in certain settings, solid tumours may yet prove to be responsive to radioimmunotherapy—particularly to the improved indirect pre-targeted methods. Radioimmunotherapy with first-generation conjugates are effective in the management of lymphomas, but adoption of the new modality in patients with nhl has been challenged by the unprecedented success of a single antibody, rituximab, especially in combination with chemotherapy 1,2,26,27. However, the current state of therapy in most solid cancers does not provide a similar advantage—particularly with such treatment-refractory neoplasms as lung, pancreatic, biliary, hepatocellular, and hormone-resistant prostatic carcinomas—and so any improvement in response attributable to radioimmunotherapy, either alone or combined with drugs or non-cytotoxic immunotherapy, would be welcome.

Other evidence indicates that any tumour will respond if given enough radiation, but the challenge, as in chemotherapy, is to make irradiation tumour-selective, sparing normal organs from toxicity. In terms of delivering radiation or even drugs, the best prospect is to conjugate them to selectively targeting antibodies, as discussed elsewhere 1,28,29. Unfortunately, because most phase i and ii trials with new agents, including radioimmunoconjugates, involve patients with refractory and advanced disease—an application that is contrary to the paradigm that the efficacy of radioimmunotherapy is inversely related to size of the target tumours 4–7,30,31—the literature is replete with failed efforts to show tumour shrinkage with one or even more administrations of radioimmunoconjugates. As clinical testing moves to address minimal disease, compartmental therapy, and the improved delivery achieved with pre-targeting strategies, I remain optimistic that the prospects for cancer radioimmunotherapy will improve.

## Figures and Tables

**TABLE I:** Approved or investigational radiolabelled antibodies for the treatment of cancer^a^

*Indication*	*Agent*
Hematologic malignancies	
Non-Hodgkin lymphoma	^90^Y–Ibritumomab tiuxetan^b^ ^131^I–Tositumomab^b^ ^90^Y–Epratuzumab anti-CD22 IgG
T-Cell lymphomas, non-Hodgkin and Hodgkin lymphomas	^90^Y–Anti-Tac IgG
Leukemia	^131^I–BC8 anti-CD45 IgG ^213^Bi–HuM195 anti-CD33 IgG ^188^Re– or ^90^Y–anti-CD66 IgG
Solid malignancies	
Colorectal cancer	^90^Y–T84.66 anti-cea IgG ^131^I– and ^90^Y–labetuzumab (anti-cea IgG) ^125^I– and ^131^I–A33 IgG ^131^I–CC49-ΔCH2 ^90^Y–Biotin pre-targeted by CC49–StAv fusion protein
Ovarian cancer	^177^Lu– and ^90^Y–CC49 ^131^I–Anti-cea IgG ^90^Y–Biotin pre-targeted by biotinylated mAb cocktail ^90^Y–Hu3S193
Prostate cancer	^177^Lu–J591 IgG
Pancreatic cancer	^90^Y–PAM4 IgG
Lung cancer	^131^I–chTNT
Hepatocellular cancer	^131^I–Hepama-1 IgG ^90^Y–hAFP IgG
Renal cancer	^131^I–cG250 IgG
Breast cancer	^90^Y–BrE3
Glioma	^131^I–81C6 antitenascin ^211^At–81C6 ^90^Y–BC2 and BC4 antitenascin ^90^Y–Biotin pre-targeted by biotin–BC4 ^125^I–425 IgG
CNS or leptomeningeal cancer	^131^I–8H9 IgG
Medulloblastoma	^131^I–3F8 IgG
Head and neck cancers	^86^Re–Bivatuzumab IgG
Medullary thyroid cancer	^131^I–Hapten pre-targeted by anti-cea bsmAb

a Adapted from Goldenberg *et al.* 17, with permission of the publisher.

b Approved by the U.S. Food and Drug Administration; all others are under investigation.

IgG = immunoglobulin G; cea = carcinoembryonic antigen.
